# Evidence of Morphological and Morphometric Differences in the Sella Turcica of *Pteronotus mesoamericanus* and *P. mexicanus*

**DOI:** 10.3390/ani15040519

**Published:** 2025-02-12

**Authors:** M. A. Peralta-Pérez, M. Briones-Salas

**Affiliations:** 1 Centro Interdisciplinario de Investigación para el Desarrollo Integral Regional Unidad Oaxaca, Instituto Politécnico Nacional, Hornos 1003, Col. Nochebuena, Santa Cruz Xoxocotlán, Oaxaca 71230, Mexico; 2 Facultad de Sistemas Biológicos e Innovación Tecnológica, Licenciatura en Biología, Universidad Autónoma Benito Juárez de Oaxaca, Av. Universidad S/N Ciudad Universitaria, Oaxaca 68120, Mexico

**Keywords:** cranial anatomy, dorsum sellae, cryptic species, pituitary gland, microchiropterans, Mormoopidae, macrophotography

## Abstract

Morphological modifications are a potential mechanism for functional species and phylogenetic diversification. The *sella turcica* in mammals is a structure associated with the basisphenoid bone and serves as the receptacle for the pituitary gland; however, little is known about the morphological variation that may affect functionality in chiropterans. In this study, we provide morphological and morphometric evidence of differences between populations of *Pteronotus mesoamericanus* [the Gulf of Mexico] and *P. mexicanus* [the Pacific Coast] by describing variations in the dimensions of the *dorsum sellae* and the *processus clinoideus caudalis* of the *sella turcica*. We obtained 20 a priori designed measurements of the *dorsum* of the *sella turcica* from 243 skulls of both species from various locations in Mexico. The *dorsum sellae* were found at an average distance of 3.4 mm from the lower edge of the *foramen magnum*. The *dorsum* of the *sella turcica* has a truncated pyramidal shape, with the *processus clinoideus caudalis* located at the tip of the pyramid. Ten of the measurements obtained were found to be significant for both regions (the Pacific Coast and the Gulf of Mexico). We propose that these measurements be tested in future studies of populations from the Mormoopidae family in the Antilles, Central America, and South America for comparative purposes, and to help distinguish different lineages and functions.

## Simple Summary

Minor modifications in the structure of living beings are signs of the gradual formation of new species. In addition, studying these changes gives us scientific tools, for example, to know whether the species of one region are different from those of another area. In this work, we aim to show these subtle differences in the cranial characteristics in the populations of bats with extensive distributions in Mexico. We now know that there are differences in the populations of *Pteronotus mesoamericanus* of the Gulf of Mexico *and P. mexicanus* of the Pacific.

## 1. Introduction

The taxonomy of mammals has developed around the morphology and morphometry of their skulls and, consequently, their phylogenetic relationships. Kinship is defined based on the shape and dimensions of the skull. It establishes correlations with the environment and possible preferences in dietary habits and other aspects. Furthermore, the skull is subject to phenotypic modifications due to natural selective interbreeding [1,2,3]. Therefore, modifications in morphological characteristics are a potential mechanism for the functional and phylogenetic diversification of species [4].

The *sella turcica* in mammals, a structure associated with the basisphenoid bone (*os basisphenoidale*), plays a significant role. Its variations, particularly in the shape and dimensions of the dorsum sellae, are of great interest. The anterior part presents the tubercle *sellae*, and continuing towards the middle part is the hypophyseal or pituitary fossa (*fossa hypophysialis*), which in a non-pathological state houses the pituitary gland. The posterior part contains the dorsum sellae, with the posterior clinoid processes (*processus clinoideus caudalis*) [5].

While the *sella turcica*’s role as the pituitary gland’s receptacle is known in humans, its functionality in chiropterans remains a mystery. This structure is known to undergo modifications in its shape according to various pituitary gland pathologies and modifications related to anatomical and dental conditions in humans [6]. Therefore, future studies should focus on unraveling the *sella turcica*’s functionality in chiropterans, particularly its potential relationship with the pituitary hormone levels in some bat species.

*Pteronotus* is widely distributed from western and southeastern Mexico, passing through Central America to Brazil. In Mexico, five species are within the genus: *Pteronotus davyi*, *P. personatus*, *P. gymnonotus*, *P. mesoamericanus*, and *P. mexicanus*, with the last two species considered subspecies of *P. parnellii*, *P. p. mesoamericanus*, and *P. p. mexicanus* [7]. Genetic studies show that there are different lineages of *P. parnelli* across its distribution range, and they propose that the subspecies *mexicanus y mesoamericanus* be recognized at the species level [8,9,10].

The identification of cryptic species, such as those in the genus *Pteronotus*, has been a significant achievement. These species, with their subtle differences, have been identified using molecular and genetic tools, bioacoustics, behavior, and ecological requirements [11,12,13]. Therefore, further evidence supporting the differentiation of these cryptic species will provide valuable insights into the evolutionary processes across the various populations within the distribution range of *Pteronotus*.

The variations in the shape and dimensions of the dorsum sellae in microchiropterans, particularly in *P. mexicanus* and *mesoamericanus*, and their relationship with the environment are crucial. These variations could potentially be applied to differentiating populations of the species, establishing the significance of these populations in evolutionary, ecological processes, and most importantly, conservation strategies for the species. This underscores the urgency and importance of our work in understanding and preserving these species.

This article aims to provide morphological and morphometric evidence of the differences among the cryptic species *P. mexicanus* and *mesoamericanus*. By describing the variations in the dimensions of the dorsum sellae and the *processus clinoideus caudals* of the *sella turcica* across different collection sites in Mexico, we hope to contribute significantly to the understanding of these species and their conservation.

## 2. Materials and Methods

A search for information was conducted without chronological restrictions of the specialized literature on the skulls of *Pteronotus mexicanus*, *mesoamericanus*, and other chiropterans to verify whether there are any records of variations in the structures described herein in national or international publications in Web of Science, Google Scholar, Latindex, and SciELO, using the following keywords in both Spanish and English: silla turca, *sella turcica*, morphology, cranial anatomy, and basicranial anatomy, all in combination with the terms bat, chiropteran, the species name, its synonyms, and the name of the family Mormoopidae.

### 2.1. Specimen Review

**Figure 1:**
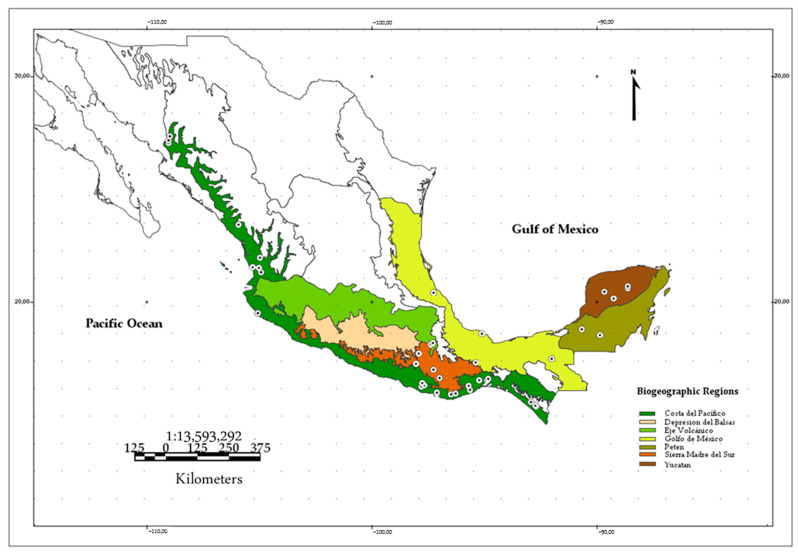
Localities where specimens of both species of *Pteronotus* were recorded.

Twenty measurements designed for the *sella turcica* were taken from 243 skulls of *P. mexicanus* and *mesoamericanus*, including 105 male and 138 female specimens. The collection sites for these specimens cover most of the species distribution area in Mexico (Figure 1; Appendix A Table A1). Additionally, to verify whether a developed or at least distinguishable *dorsum sellae* exists and its shape in other bat species, the skulls of 25 other chiropteran species were examined (although no measurements of the *dorsum* were made for these species). This review included three species phylogenetically related to *P. mexicanus* and *mesoamericanus: P. fulvus*, *P. psilotis*, and *Mormoops magalophylla*.

The 243 specimens were organized by region within Mexico, resulting in 150 speci-mens from the Mexican Pacific region and 77 specimens from the Gulf of Mexico region. For this analysis, specimens from the Petén and Yucatán regions were included in the Gulf of Mexico region analysis. Specimens from the Sierra Madre del Sur and Eje Volcáni-co regions were excluded from the analysis due to sample size limitations. They have not yet undergone a genetic analysis that distinguishes them according to one of the two populations proposed by López-Wilchis et al. [8]. However, their measurement values are used to assign them to one of the two species.

The museum specimens analyzed are in two scientific collections that house significant numbers of specimens of the species: the Regional Collection of Mammals of the Interdisciplinary Center for Regional Research and Development, the Oaxaca Unit, of the National Polytechnic Institute (OAX.MA.026.0497), and the Collection of the Center for Biological Research of the Northeast, S.C. (CIBNOR).

### 2.2. Photographs

**Figure 2:**
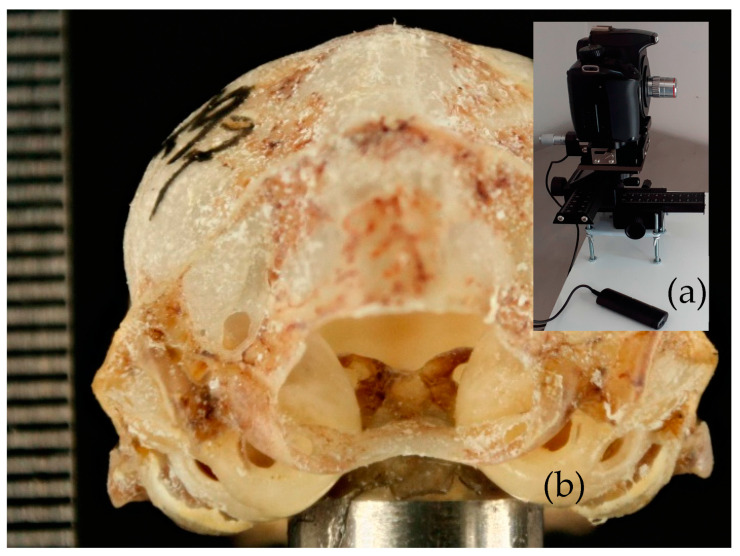
(**a**) Device for obtaining macrophotography and (**b**) occipital view of a *Pteronotus* skull mounted on a metal rod. The focus was on the interior through the foramen magnum.

Images were taken using a CANON EOS REBEL XS^®^ camera with an RMS^®^ microscope objective adapter, to which an AmScope 4X Planachromatic^®^ objective was attached. The camera was mounted on a Zouminy^®^ micrometer screw stage and placed on a four-way sliding control rail for macrofocusing by Bewinner^®^, with all components fixed to a steel plate separated from the work surface by synthetic rubber to reduce vibration. Each *P. mexicanus* and *mesoamericanus* skull was mounted on a stem inside a lightbox for photography and positioned in a front-occipital view towards the objective lens, focusing the interior through the *foramen magnum* in orthogonal projection (Figure 2). The anatomical terminology used to describe the structure followed the Veterinary Anatomical Nomenclature [14].

### 2.3. Measurements

**Figure 3:**
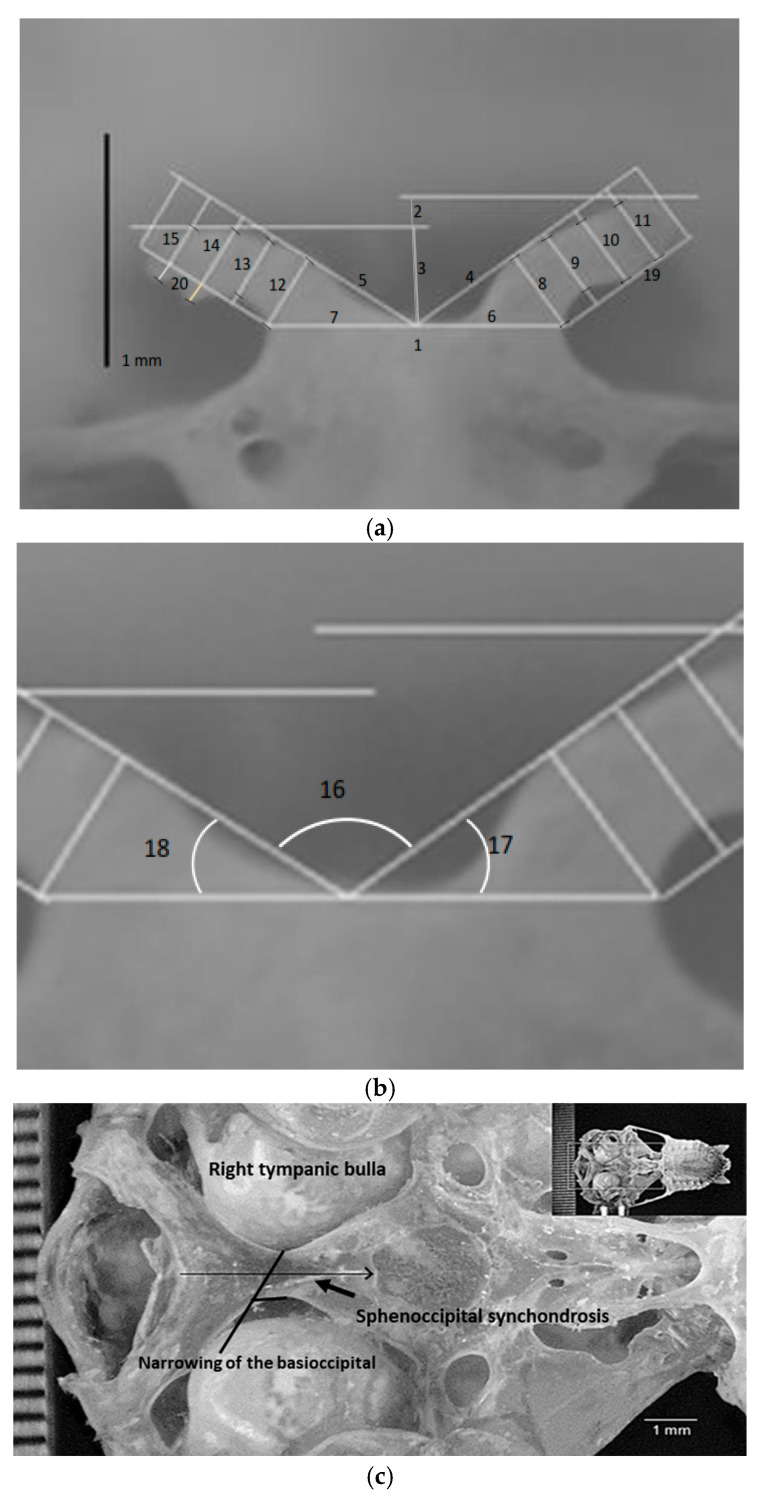
Schematic of recorded lengths and angles of the *dorsum sellae* and *processus clinoideus caudalis* (**a**,**b**) indicating where the *dorsum sellae* is the edge of the *foramen magnum* in the ventral view of the skull (the thin black arrow indicates the site of the synchondrosis) (**c**). For a description of the measurements, see Table 1.

Ad hoc measurements were to be reproducible for the dorsum sellae and the *processus clinoideus caudalis*. The measurements were taken using ImageJ software, version 1.53k [15], with a scale attached to the image. A diagram of the 15 measured lengths and three angles, along with their descriptions, is shown in Figure 3, Table 1.

A Mann–Whitney U test analysis was applied using SPSS V.21 [16] to identify differences between the distributions of the Pacific and Gulf of Mexico populations.

## 3. Results

### 3.1. General Description

The morphological variation in the *sella turcica* in *P. mexicanus* and *mesoamericanus* is a comprehensive study, present in all 243 skulls examined and measured. It starts from the floor of the skull, formed by the basioccipital bone, which is generally broad in mammals and is between the tympanic bullae. Uniquely, the basioccipital bone in the species under study narrows forward (Figure 3c) until it is as narrow as less than half of the proximal end to the foramen magnum.

The described structure is in the occipital view through the foramen magnum. The *dorsum* of the *sella* and its caudal clinoid processes are in the spheno-occipital joint. From the occipital view, the *sella turcica* is by the auditory bullae. In the ventral view, the *sella turcica* is after the basioccipital fossa in an anteroposterior direction (Figure 2).

Regarding the skulls reviewed and photographed from other bat species, in addition to *P. mexicanus* and *mesoamericanus*, only members of its family [Mormoopidae] exhibited conspicuous *dorsum sellae* and *processus clinoideus caudalis*. In contrast, two species from the Phyllostomidae family, *Artibeus lituratus* [frugivore] and *Glossophaga soricina* [nectarivore/pollinivore], have small protrusions that are barely perceptible under a stereoscopic microscope (Table 2). The *dorsum sellae* is truncated pyramidal, with the *processus clinoideus caudalis* at the pyramid’s apex.

### 3.2. Morphometry

In *P. mexicanus* and *mesoamericanus*, spheno-occipital synchondrosis is present. Therefore, the *dorsum sellae* is at an average distance of 3.4 mm [ranging from 3.25 to 3.5 mm, with a standard deviation of 0.12 mm] from the lower border of the *foramen magnum* (Figure 3c and Table 3).

**Figure 4:**
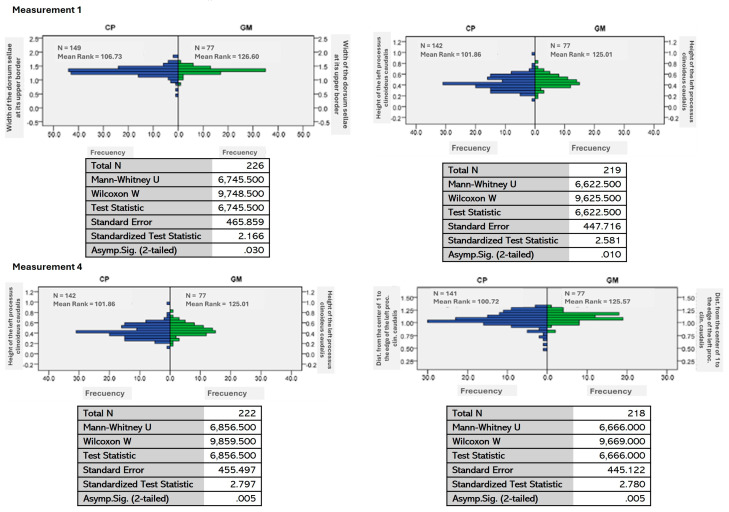

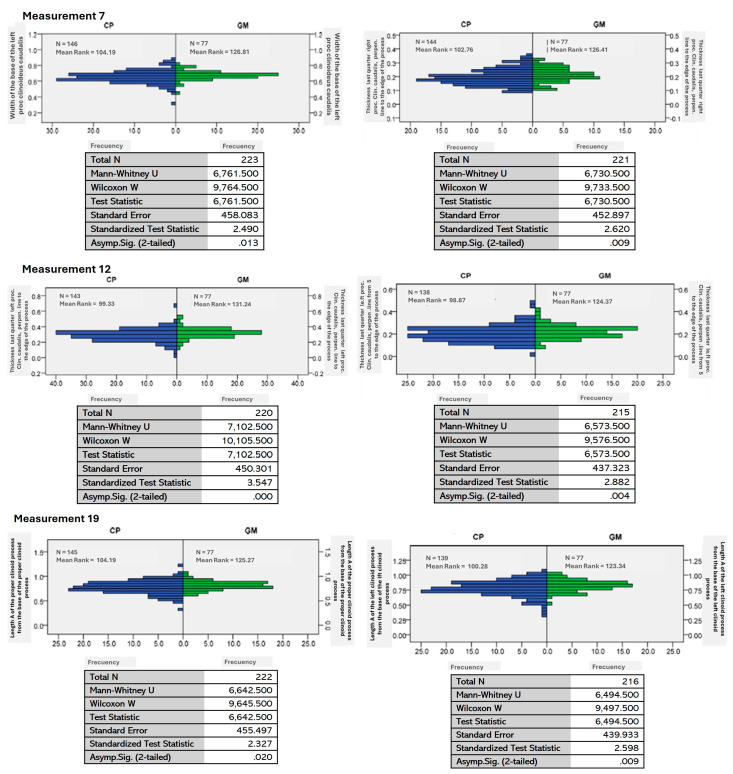
Graphics of independent-sample Mann–Whitney U test for ten measurements: the Coastal Pacific (CP, in blue) vs. Gulf of Mexico (GM, in green) regions.

A Mann–Whitney U test for independent samples was conducted at an asymptotic significance level of 0.05. This test rejected the null hypothesis in ten measurements taken from the *sella turcica*, indicating significant differences between the samples from the biogeographic zones known as the Pacific Coast and the Gulf of Mexico [Figure 4, Appendix A Table A2].

Regarding the specimens from Sierra Madre del Sur and the Volcanic Axis, a visual comparison of their measurements versus the average, maximum, and minimum values of each of the regions analyzed achieved the definition that one is within the intervals of the Pacific Coast region with 15 measurements that correspond to the species *Pteronotus mexicanus* [measurements 2, 3, 4, 8, 9, and 11–20]; two specimens with 12 measurements are within the intervals of *P. mesoamericanus* [measurements 1,2, 3, 4, 6, 7, 8, 11, 12, 17, 19, and 20]; and finally, the remaining specimens were not defined as belonging to either of the two species.

## 4. Discussion

Regarding the literature review of books and scientific journals, there were no records concerning the structural variation in the *sella turcica* as described here for the genera of the Mormopidae family. According to Velazco’s [17] review of the *sella turcica* in other bat species, *Carollia subrufa*, *Sturnira erythromos*, and *Uroderma magnirostrum* do not possess either the *dorsum* or these processes. In contrast, the genera *Platyrrhinus* and *Vampyrodes* present small, almost imperceptible clinoid processes; however, the *dorsum sellae* is small and undetectable. There are no measurements in the cited work.

**Figure 5:**
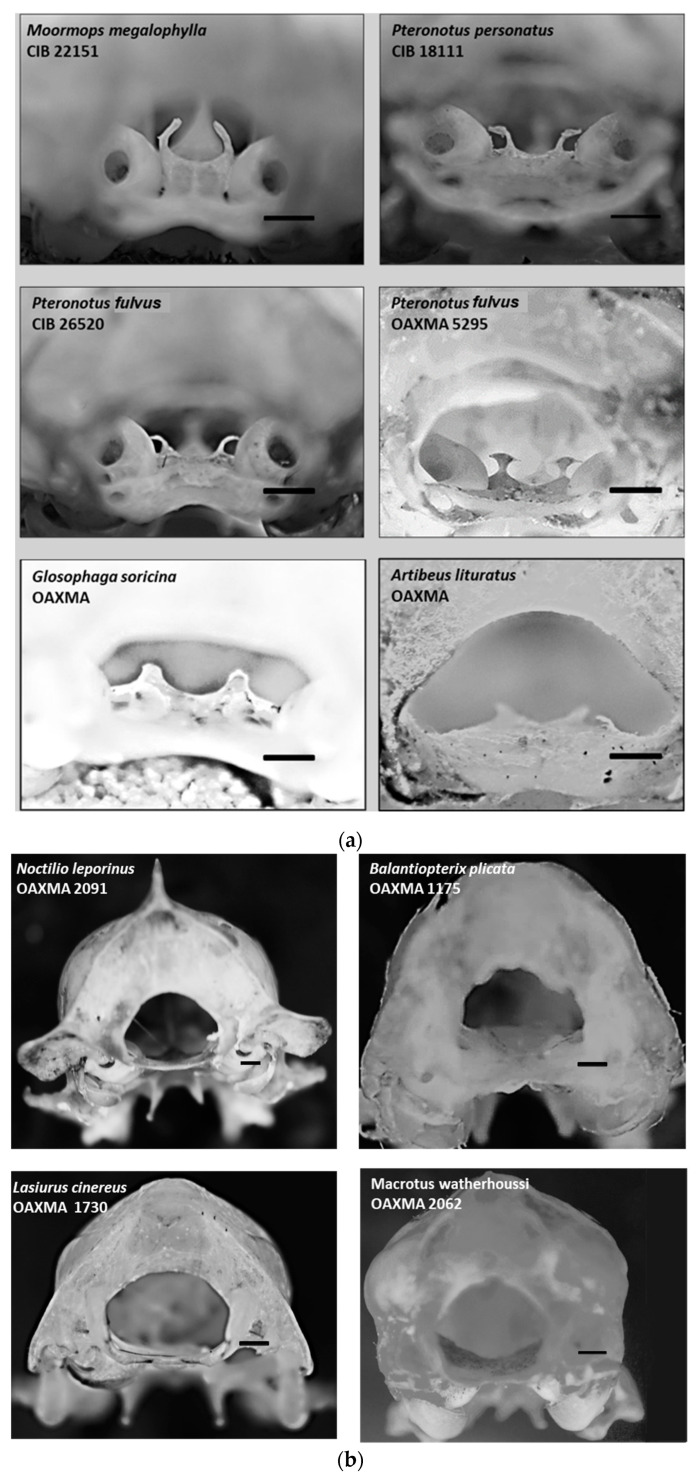
(**a**) Images of the dorsum sellae and posterior clinoid processes in the skulls of other species of the Moormopidae family (*Moormops megalophylla*, *P. fulvus*, *P. psilotis*, and other microchiropterans *Glossofaga soricina* and *Artibeus lituratus*). (**b**) Images of the occipital view in which there is an absence of the dorsum sellae and its clinoid processes in the skulls of *Noctilio leporinus*, *Baliantiopterix plicata*, *Lasiurus cinereus*, and *Macrotus waterhoussi*. The black line is equivalent to 1 mm.

Based on the specimens reviewed, only in the Mormoopidae family does the *sella turcica* possess an elevated *dorsum sellae* above the floor of the skull with prominent clinoid processes. The posterior shape of the *sella turcica* is evident to the observer, much like in other mammals [e.g., Carnivora]. In contrast, in the other bat genera reviewed, the *dorsum sellae* of the *sella turcica* is completely reduced. In some cases, only discrete protuberances are discernible, with the area of the spheno-occipital synchondrosis where the *sella turcica* is being practically smooth [Table 2 and Figure 5a,b].

The description of the basisphenoid provided by Simmons and his collaborators [18] indicates that this bone narrows to one-third of the width of the *foramen magnum*, the *dorsum* of the *sella turcica* being at that narrowest site of the basisphenoid. It is likely that the growth of tympanic bullae, mainly located deep within the skull of the family Mormoopidae, causes the narrowing of the width of the basilesphenoid.

From this sample, we can affirm that the presence of the *dorsum* of the *sella turcica* in the Mormoopidae family is conspicuous, and its morphology is typical of any mammal. In *Pteronotus mexicanus* and *mesoamericanus*, there are various shapes across all populations, and their dimensions vary among populations. Notably, the populations of the Pacific region and those from the Gulf of Mexico region show apparent differences. According to the hypothesis of the family origin, both populations stem from groups that migrated along different routes to the continent from the Antilles. Therefore, this structure should be like the species of this family in the Antilles.

The *dorsum* of the *sella turcica* and the clinoid processes in *P. mexicanus and mesoamericanus* show a wide range of dimensions in the recorded localities in Mexico, where each region has very different environmental conditions. The growth and shape of the *sella turcica dorsum* may result from pituitary-level responses and, thus, adaptations to the various climates, types of vegetation, and diets of this species within its distribution range in the country. This is supported by the location of the *sella turcica* in the sellar region. This small area also includes the cavernous sinus and the suprasellar cistern. It houses the pituitary gland, making this region crucial for neurovascular processes and endocrine and optical functions [19].

While our study suggests a potential sympatry zone between *P. mexicanus* and *mesoamericanus*, indicated by the specimens from localities in the Sierra Madre del Sur and the Volcanic Axis, it is important to note that this finding is subject to the reservations that the deficiencies of the sample impose. These reservations are crucial to consider for the credibility of our research.

The hormonal activity in the *sella turcica* area is intense, as it contains the pituitary gland. The posterior lobe of the pituitary is mainly regulated by neurohypophysis, which has axonal projections from hypothalamic neurons [pituicytes], and their endings, known as Herring bodies, serve as hormone reservoirs, such as the antidiuretic hormone [ADH] and oxytocin. This activity is related to changes in the shape and dimensions of the various components of the *sella turcica* [20,21,22]. Therefore, we consider it important to investigate the hormonal linkage of the shape of the *sella turcica* in the Mormoopidae family.

In humans, the dimensions of the *sella turcica*, including its length and diameter, increase with age. This growth rate slows in preschool and then increases considerably during puberty [23]. Afterward, it decreases and eventually ceases at the onset of adulthood. Therefore, the different shapes and dimensions recorded in the collection localities may be related to the ontogenetic development of the mammal species studied here.

This characteristic, consistently present in the skulls of terrestrial mammals but not in flying ones, could be considered a plesiomorphic trait in both species and the Mormoopidae family, potentially signaling their isolation in the past on the Caribbean islands [8].

The measurements for the *dorsum* of the *sella turcica* in *P. mexicanus and mesoamericanus* were explicitly used for this study. For comparative purposes, these measurements should be used in future studies of Mormoopidae species populations from the Antilles, Central America, and South America.

## 5. Conclusions

This pioneering work unveils, for the first time, the unique characteristics of the *dorsum sellae* in the skull of a microchiroptera.

The data gathered here not only confirm the existence of differences in the measurements of the dorsum sellae of the populations of the Gulf and Pacific regions in Mexico but also provide support for the division of *P. parnelli* into two species: *P. mesoamericanus* [the Gulf region] and *P. mexicanus* [the Pacific region]. It is crucial to bear in mind the limitations of the sample when interpreting the findings of this study.

## Figures and Tables

**Table 1:** Description of the measurements used for the dorsum sellae and *processus clinoideus caudalis*.

No.	Description of Measurement
1	Width of the *dorsum sellae*;
2	Height of the right *processus clinoideus caudalis*;
3	Height of the left *processus clinoideus caudalis*;
4	Distance from the center of measurement 1 to the edge of the right *processus clinoideus caudalis*; the line must touch the edge of the clinoid process;
5	Distance from the center of the width of the *dorsum sellae* to the edge of the left *processus clinoideus caudalis*;
6	Width of the base of the right *processus clinoideus caudalis*;
7	Width of the base of the left *processus clinoideus caudalis*;
8	Thickness of the first quarter of the right *processus clinoideus caudalis*;
9	Thickness of the second quarter of the right *processus clinoideus caudalis*;
10	Thickness of the third quarter of the right *processus clinoideus caudalis*;
11	Thickness of the last quarter of the right *processus clinoideus caudalis*;
12	Thickness of the first quarter of the left *processus clinoideus caudalis*;
13	Thickness of the second quarter of the left *processus clinoideus caudalis*;
14	Thickness of the third quarter of the left *processus clinoideus caudalis*;
15	Thickness of the last quarter of the left *processus clinoideus caudalis*;
16	Angle between the lines of measurements 4 and 5;
17	Angle between the lines of measurements 4 and 6;
18	Angle between measurement lines 5 and 7;
19	Length A of the proper clinoid process from the base of the proper clinoid process;
20	Length A of the left clinoid process from the base of the left clinoid process.

**Table 2:** Species reviewed in the Regional Mammal Collection and the Northeast Biological Research Center, S.C., verified the presence of the *dorsum sellae* or the *processus clinoideus caudalis* in different species (P: present; N: not present).

Species	Presence	Species	Presence
*Pteronotus fulvus*	P	*Rhogeesa parvula*	N
*Pteronotus psilotis*	P	*Lasiurus frantzii*	N
*Mormoops megalophylla*	P	*Aeroestes cinereus*	N
*Macrotus waterhousii*	N	*Plecotus mexicanus*	N
*Balantiopterix plicata*	N	*Corynorhinus townsendii*	N
*Noctilio leporinus*	N	*Eptesicus fuscus*	N
*Tadarida brasiliensis*	N	*Myotis californicus*	N
*Molossus rufus*	N	*Myotis keysi*	N
*Nyctinomops macrotis*	N	*Myotis thysanodes*	N
*Promops centralis*	N	*Myotis vellifer*	N
*Molossus molosus*	N	*Myotis nigricans*	N
*Rhogeesa alleni*	N	*Glossophaga soricina*	P
*Artibeus lituratus*	P		

**Table 3:** Values of the *sella turcica* measurements of *Pteronotus mexicanus* and *mesoamericanus* for all specimens [measurements in mm] [n = 243].

Measure	Average	Minimum Value	Maximum Value	Standard Deviation	Variance	Significance
1	1.32	0.48	1.82	0.15	0.02	0.03
2	0.42	0.11	1.006	0.12	0.015	0.01
3	0.44	0.12	0.96	0.12	0.01	0.01
4	1.07	0.47	1.52	0.12	0.01	0.01
5	1.07	0.46	1.34	0.13	0.01	0.01
6	0.66	0.30	0.95	0.07	0.005	
7	0.66	0.30	0.89	0.07	0.005	0.01
8	0.29	0.07	0.5	0.07	0.005	
9	0.21	0.08	0.39	0.04	0.002	
10	0.21	0.07	0.38	0.05	0.003	
11	0.20	0.09	0.37	0.05	0.003	0.01
12	0.29	0	0.65	0.07	0.006	0.00
13	0.21	0	0.34	0.05	0.003	
14	0.21	0.004	0.44	0.06	0.004	
15	0.20	0.004	0.47	0.07	0.004	0.004
16	119.04	26.96	180	15.10	228.01	
17	29.86	12.13	50.66	7.17	51.53	
18	30.92	0.31	61.98	7.33	53.80	
19	0.78	0.33	1.22	0.12	0.01	0.02
20	0.79	0.31	1.08	0.12	0.01	

## Data Availability

Further inquiries can be directed to the corresponding authors.

## Appendix A

**Table A1:** The numbers of specimens for the localities used in this study and their geographic locations.

Locality	State	Biogeographic Regions (BIOGEO)	Lat.	Lon.	N
Chamela	Jalisco	Costa del Pacífico	19.5	−105.04	24
Compostela	Nayarit	Costa del Pacífico	21.31	−104.9	2
El Venado	Nayarit	Costa del Pacífico	21.96	−104.95	1
San Blas	Nayarit	Costa del Pacífico	21.54	−105.28	2
Tepic	Nayarit	Costa del Pacífico	21.51	−104.99	1
La Venta, Santo Domingo Ingenio	Oaxaca	Costa del Pacífico	16.53	−94.84	4
Magdalena Tacotepec	Oaxaca	Costa del Pacífico	16.53	−95.2	2
Pluma Hidalgo	Oaxaca	Costa del Pacífico	15.9	−96.45	1
San Francisco Huamelula	Oaxaca	Costa del Pacífico	16.1	−95.61	1
San José de las Flores	Oaxaca	Costa del Pacífico	16.4	−97.73	2
San Pedro Mixtepec	Oaxaca	Costa del Pacífico	15.92	−97.08	12
Santa María Petatengo	Oaxaca	Costa del Pacífico	15.94	−96.03	6
Santiago Jamiltepec	Oaxaca	Costa del Pacífico	16.24	−97.77	1
Santiago Tetepec	Oaxaca	Costa del Pacífico	16.3	−97.65	2
Copala	Sinaloa	Costa del Pacífico	23.42	−105.91	25
Álamos	Sonora	Costa del Pacífico	27.04	−109.01	40
Mina San Alberto, Mexiquillo	Sonora	Costa del Pacífico	27.35	−108.96	18
Constitución Mexicana	Oaxaca	Golfo de México	17.31	−95.35	1
Estación Los Tuxtlas	Veracruz	Golfo de México	18.59	−95.08	4
Chichen-itza	Yucatán	Yucatán	20.6	−88.59	7
Ticul-Muna	Yucatán	Yucatán	20.45	−89.63	14
Ticum	Yucatán	Yucatán	20.15	−89.22	11
Ayoquezco de Aldama	Oaxaca	Sierra Madre del Sur	16.67	−96.87	1
San Juan Mazatlán	Oaxaca	Sierra Madre del Sur	17.71	−97.89	1
San Miguel Cuevas	Oaxaca	Sierra Madre del Sur	17.26	−98.01	3
San Miguel Piedras	Oaxaca	Sierra Madre del Sur	17	−97.23	2
San Sebastian de las Grutas	Oaxaca	Sierra Madre del Sur	16.62	−96.95	7
Tepelmeme, Villa de Morelos	Oaxaca	Sierra Madre del Sur	18.12	−97.36	1
San José Miahuatlán	Puebla	Eje volcánico	18.17	−97.26	1
Number of individuals					243

**Table A2:** Summary of the Mann–Whitney U hypothesis test for independent samples showing only the measurements where the null hypothesis is rejected.

Measures	Null Hypothesis	Significance
1	The distribution of the dorsum width is the same among BIOGEO categories.	0.030
3	The distribution of the perpendicular height of the left clinoid process is the same among BIOGEO categories.	0.010
4	The distribution of the length of the right clinoid process from the center of width 1 is the same among BIOGEO categories.	0.005
5	The length distribution of the left clinoid process from the center of the width is the same among BIOGEO categories.	0.005
7	The distribution of the base of the left clinoid process is the same between BIOGEO categories.	0.013
11	The width 11 distribution of the right clinoid process is the same between BIOGEO categories.	0.009
12	The width 12 distribution of the left clinoid process is the same between BIOGEO categories.	0.000
15	The width 15 distribution of the left clinoid process is the same between BIOGEO categories.	0.004
19	The length 19 distribution of the right clinoid process from the base of the right clinoid process is the same between BIOGEO categories.	0.020
20	The length 20 distribution of the left clinoid process from the base of the left clinoid process is the same between BIOGEO categories.	0.009
